# Misdiagnosis of gestational trophoblastic neoplasia as ectopic pregnancy: A 15-year retrospective study

**DOI:** 10.3389/fmed.2022.1018573

**Published:** 2022-11-02

**Authors:** Ping Xiao, Tao Guo, Rutie Yin

**Affiliations:** Ministry of Education Key Laboratory of Birth Defects and Related Diseases of Women and Children, Department of Obstetrics and Gynecology, West China Institute of Women and Children's Health, West China Second University Hospital, Sichuan University, Chengdu, China

**Keywords:** gestational trophoblastic neoplasia, ectopic pregnancy, human chorionic gonadotropin, misdiagnosis, retrospective study

## Abstract

**Background:**

Gestational trophoblastic neoplasia is an uncommon disease, whose clinical manifestations are similar to ectopic pregnancy, thus some rare pelvic lesion can be misdiagnosed as ectopic pregnancy.

**Aims:**

This study was presented to investigate the characteristics of gestational trophoblastic neoplasia misdiagnosed as ectopic pregnancy and reduce the misdiagnosis.

**Methods:**

The clinicopathological data for 14 cases of gestational trophoblastic neoplasia misdiagnosed as ectopic pregnancy at West China Second Hospital Sichuan University from January 2006 to December 2020 were retrospectively analyzed.

**Results:**

The main clinical manifestations were amenorrhea, abnormal vaginal bleeding, and abdominal pain. At initial diagnosis, the serum hCG level was >10,000 mIU/mL in 5 patients and <10,000 mIU/mL in 7 patients, and a positive urine pregnancy test alone was found in 2 patients. Vaginal ultrasonography showed no abnormalities in 7 cases, adnexal mass in 5 cases, and tubal thickening in 2 cases. The patient's previous pregnancy was an abortion in 7 cases, full-term in 4 cases, and a hydatidiform mole in 3 cases. Clinical stage: 3 cases were stage I, 3 were stage II, 7 were stage III, and 1 case was stage IV (liver and spleen metastases). The median FIGO prognostic score was 13.5 points (12–21 points), with 9 cases having a score >13 points (very high risk). From 14 patients, only 3 had molar pregnancy previously. Only 3 patients had no metastasis at GTN diagnosis (from these 3, only one after molar pregnancy). After chemotherapy alone or in combination with surgery, all patients survived, with a median follow-up of 84 months (23–102 months).

**Conclusion:**

If we have positive hCG, without a sonographic topic gestation confirmation, associated with metastatic lesions, the GTN diagnosis should be considered instead of ectopic pregnancy, if the patient have had a pregnancy once during her life.

## Introduction

Gestational trophoblastic disease (GTD) is a group of diseases caused by the abnormal proliferation of trophoblast cells in the placenta, which include benign mole and malignant trophoblast diseases. According to the WHO (5th Edition, 2020) pathological classification of the female reproductive system (1), GTD can be histologically divided into: (1) gestational trophoblastic neoplasia (GTN), including choriocarcinoma, placental site trophoblastic tumors (PSTT), epithelioid trophoblastic tumors (ETT), and mixed trophoblastic tumors; (2) hydatidiform mole pregnancy, including complete, partial, and invasive/metastatic hydatidiform mole; (3) tumor-like lesions (arterial-like lesions), including abnormal placental reactions and placental nodules/plaques; and (4) abnormal (non-hydatidiform mole) villous lesions. GTN can be secondary to any type of pregnancy, but has a low incidence ranging from 1–3/1,000 pregnancies for hydatidiform mole to (1–9)/40,000 pregnancies for choriocarcinoma (2).

The clinical manifestations of GTN include amenorrhea, abnormal vaginal bleeding, and elevated levels of serum human chorionic gonadotropin (hCG) in most patients, and these are also common clinical features in ectopic pregnancy. As only about 50% of GTN follows molar pregnancy, the rest can occur after an abortion, ectopic pregnancy, or a term pregnancy. Aside from abnormal vaginal bleeding, other clinical presentations may include bleeding from metastatic sites such as the lung, or brain, liver, spleen, intestines (3).

The incidence of ectopic pregnancy (EP) is ~1–2% (4). In particular, GTN patients with extrauterine lesions are more likely to be misdiagnosed with ectopic pregnancy. GTN is mainly treated with chemotherapy, while EP is treated with surgery or drug therapy, and misdiagnosis will lead to an incorrect treatment strategy for patients. The present paper reviews the clinicopathological data for GTN patients admitted to our hospital over the past 15 years, analyzes the clinical characteristics of patients misdiagnosed with EP, reviews the literature, and discusses the causes of misdiagnosis, with a view to facilitating correct clinical diagnosis and reducing misdiagnosis.

## Materials and methods

Clinicopathological data for GTN patients misdiagnosed with ectopic pregnancy, who were admitted to the West China Second Hospital of Sichuan University from January 2006 to December 2020, were collected. These included age, clinical manifestations, main treatment course after misdiagnosis, previous pregnancy type prior to the diagnosis of GTN, time since the previous pregnancy, serum hCG levels before treatment, International Federation of Obstetrics and Gynecology (FIGO) prognosis score and stage (2000), treatment, and prognosis were summarized and analyzed to elucidate the causes of misdiagnosis and suggest preventive measures.

Due to the small sample size, the distribution of continuous variables is described as the median and range. The present study was approved by the Ethics Committee of the West China Second Hospital of Sichuan University. Informed consent was obtained from all patients.

## Results

### Clinical characteristics of misdiagnosed patients

From January 2006 to December 2020, a total of 611 patients with GTN were admitted to our hospital, among which 14 (2.29%) were misdiagnosed as ectopic pregnancy. Thirteen cases were misdiagnosed at other hospitals and 1 case was misdiagnosed at our hospital. The median age of patients in this group was 32 years old (range: 21–49 years old). Ten patients were suffering from amenorrhea, with or without vaginal bleeding and abdominal pain; and 4 patients without a clear history of amenorrhea all had vaginal bleeding, with or without abdominal pain. At the time of misdiagnosis, the serum hCG level was >10,000 mIU/mL in 5 patients and <10,000 mIU/mL in 7 patients, and a positive urine pregnancy test alone was found in 2 patients. Vaginal ultrasonography showed no obvious abnormalities in 7 cases, adnexal mass in 5 cases, and tubal thickening in 2 cases. Details are shown in Table 1.

**Table 1:** Clinical characteristics of 14 misdiagnosed patients.

**Patient**	**Age**	**Clinical manifestations**	**Serum hCG^a^ level (mIU/mL)**	**Ultrasound results**	**Treatment and outcome**
1	41	Amenorrhea, vaginal bleeding	7229.0	Right adnexal mass, 10.5 × 8.6 cm	Emergency laparoscopic exploration was performed and a 10 × 8 × 8 cm purplish-red mass was found on the right pelvic wall. Considering that the lesions did not conform to those conventional of ectopic pregnancy and the hCG level was high, so diagnosis as GTN^b^ was done and the surgery was stopped.
2	40	Amenorrhea, vaginal bleeding	3445.0	No abnormalities in or outside the uterus	No obvious lesions were found during laparoscopic exploration; MTX^c^ treatment was given, but the serum hCG was not monitored. The patient suffered from chest pain, fatigue, and dizziness 9 months following MTX treatment.
3	30	Amenorrhea, abdominal pain	2068.0	Right fallopian tube thickened	MTX was given as the initial treatment, but the serum hCG increased; bilateral laparoscopic resection of the fallopian tubes was performed, but pathological examination was inconclusive. Postoperative serum hCG continued to rise.
4	49	Amenorrhea, abdominal pain	12049.0	No abnormalities in or outside the uterus	Both fallopian tubes were resected by laparoscopy, uterine curettage was performed, and trophoblast cells were found during pathological examination. After surgery, the serum hCG was unsatisfactorily decreased, and ultrasonography suggested disorderly echoes in the uterine cavity. Uterine curettage was performed again twice, but vaginal bleeding and uterine occupation were still observed.
5	23	Amenorrhea,	2236.0	Right fallopian tube thickened	After MTX + mifepristone treatment, the serum hCG continued to rise.
6	22	Amenorrhea, vaginal bleeding	3789.0	No abnormalities in or outside the uterus	No obvious lesions were found during laparoscopic exploration; MTX + mifepristone treatment was given, but the serum hCG continued to rise.
7	28	Amenorrhea,	40890.0	Left adnexal mass, thickened	Laparoscopic exploration revealed varicose veins below the broad ligament on the left side; no abnormalities were found in either adjunct. Pathological examination indicated trophoblast cells following uterine curettage. MTX + Chinese medicine was given as treatment; the hCG decreased but vaginal bleeding continued.
8	23	Amenorrhea, vaginal bleeding	10146.0	No abnormalities in or outside the uterus	An MTX regimen was given, but the hCG continued to rise.
9	21	Vaginal bleeding	87505.0	Broad ligament mass, 3.6 × 4.5 cm	Laparoscopic resection of the broad ligament lesion was performed. Postoperative vaginal bleeding continued; pathological consultation at our hospital found trophoblast cells but no villi.
10	29	Vaginal bleeding, abdominal pain	Positive Urine hCG	Left adnexal mass, 2.8 × 3.0 cm	Laparotomy was performed and a purplish-blue lesion was found in the left ovary, which was removed. Postoperative pathological examination at the initial hospital suggested choriocarcinoma of the left ovary.
11	44	Vaginal bleeding	Positive urine hCG	No abnormalities in or outside the uterus	No obvious lesions were found during laparoscopic exploration; vaginal bleeding continued after surgery.
12	45	Amenorrhea,	507.0	No abnormalities in or outside the uterus	No abnormalities were found during exploratory laparotomy and no villi were found following curettage. The serum hCG continued to rise following treatment.
13	45	Amenorrhea, vaginal bleeding	90533.0	Left adnexal mass, 3.0 × 3.6 cm	The ovarian lesions were removed by laparoscopy; postoperative pathological examination showed ovarian follicular cysts, no villi were found in the uterus following curettage, and the serum hCG continued to rise.
14	34	Vaginal bleeding	843.0	No abnormalities in or outside the uterus	Following treatment with MTX + mifepristone, the hCG increased significantly and lesions were found in the right cornua uteri.

a hCG, human chorionic gonadotropin;

b GTN, gestational trophoblastic neoplasia;

c MTX, methotrexate.

### Treatment following diagnosis with EP

Among the 14 patients, 10 were initially treated with surgical exploration, including 1 case misdiagnosed at our hospital and 9 misdiagnosed at other hospitals. The patient misdiagnosed at our hospital was found to have massive and vascularized lesions on the lateral wall of the pelvis during laparoscopic exploration. GTN was considered, and the operation was stopped immediately. Among the 9 patients misdiagnosed at other hospitals, 5 had no intra- or extrauterine lesions detected by vaginal ultrasonography prior to surgery, and no exact lesions were found during surgery. In one case, both fallopian tubes were removed, and no abnormalities were found during the postoperative pathological examination. In the other 4 cases, the lesions all occurred at non-tubal sites: 2 ovaries, 1 broad ligament, and 1 pelvic wall. The first 3 patients received lesion resection; pathological examination indicated that one of the patients with an ovarian lesion had GTN and the other patient had an ovarian follicular cyst. No GTN features were found in the patient with broad ligament lesions. Surgery was stopped in the patient with lateral pelvic wall lesions due to the discovery of atypical lesions with tortuous vessels. Of the 9 patients misdiagnosed at other hospitals, only one (with an ovarian lesion) was corrected to a diagnosis of GTN following surgery. Among the remaining 8 patients, 3 received methotrexate (MTX) alone or in combination with mifepristone following surgery; however, the treatment was unsuccessful. The other 5 patients were transferred to our hospital due to continuously elevated serum hCG levels and/or continuous vaginal bleeding after surgery. Details are shown in Table 1.

The initial treatment in 4 patients was drug therapy, including MTX + mifepristone in 2 patients and MTX alone in the other 2 patients. Treatment failed in all 4 patients and the serum hCG continued to rise; GTN was then considered in 2 of these patients. Laparoscopic exploration was performed in one patient to remove both oviducts. Intraoperative findings and postoperative pathological examination were inconclusive. The serum hCG levels continued to rise after surgery, and GTN was considered following transfer to our hospital. The other patient received ultrasound examination again, which still showed no obvious intra- or extrauterine abnormalities; uterine curettage was subsequently performed. Postoperative pathological examination showed no GTN features, but the serum hCG levels continued to increase. GTN was considered following transfer to our hospital. Details are shown in Table 1.

### Clinical features following diagnosis with GTN

Following GTN diagnosis, 5 patients progressed from no obvious intra- or extrauterine abnormalities to suspicious lesions detected by vaginal ultrasonography, including 2 cases in the uterine cavity, 2 cases in the intramural space, and 1 case in the *cornua uteri*. When diagnosed with GTN, the patient's previous pregnancy was an abortion in 7 cases, full-term in 4 cases, and a hydatidiform mole in 3 cases. The median interval from the first pregnancy to a diagnosis of GTN was 17.5 months (2–252 months), with the interval in 9 cases being >12 months. The median serum hCG level was 6077.5 mIU/mL (230.5–634960.1 mIU/mL), with the level in 3 patients being ≥100,000 mIU/mL. The clinical stage was stage I in 3 cases, stage II in 3 cases, stage III in 7 cases, and stage IV in 1 case with liver and spleen metastasis. The median FIGO (2000) prognostic score was 8 points (3–22 points), with 2 patients having a score ≥13 points (extremely high risk). Details are shown in Table 2.

**Table 2:** The clinical characteristics, treatment, and prognosis of 14 patients diagnosed with GTN.

**Patient**	**Previous pregnancy**	**Time interval between previous pregnancy and diagnosis (months)**	**hCG^a^ level before treatment (mIU/mL)**	**FIGO^b^ Prognostic Score (2000)**	**Clinical stage (site of metastasis)**	Treatment regimen ^*^courses	**Total number of courses**	**Number of consolidation courses**	**Follow-up time (months)**	**Prognosis**
1	Full-term birth	252	1064.5	10	III	MTX^c^^*^4, KSM^d^^*^4	8	2	84	hCG negative
2	Abortion	9	200237.6	16	III	5-FU^e^+KSM^*^7, laparoscopic uterine lesion resection, followed by 5-FU+KSM^*^2	9	3	84	hCG negative
3	Abortion	19	140792.0	9	III	5-FU+KSM^*^7, followed by laparoscopic hysterectomy and subsequent 5-FU+KSM^*^1	8	2	82	hCG negative
4	Abortion	11	29578.0	5	I	EMA/CO^f^^*^3, laparoscopic total hysterectomy, EMA/CO^*^1	4	1	93	hCG negative
5	Hydatidiform mole	16	10799.7	7	III	EMA/CO^*^8	8	3	90	hCG negative
6	Abortion	7	7612.9	5	III	MTX^*^2, KSM^*^5	7	2	89	hCG negative
7	Abortion	6	230.5	6	II	MTX^*^2, KSM^*^2, EMA/CO^*^2, EMA/EP^g^^*^5	11	3	88	hCG negative
8	Full-term birth	14	634960.1	22	IV (liver and spleen metastasis)	EMA/CO^*^8 (After 4 courses of chemotherapy, the liver metastasis ruptured, and left hepatic artery embolization was performed. After another 4 courses of postoperative EMA/CO chemotherapy, hCG decreased to 75.7 mIU/mL; the treatment was stopped of the patient's own accord, and the hCG was negative 2 weeks later).	8	/	102	hCG negative
9	Abortion	2	2907.3	3	II	MTX^*^5	5	2	81	hCG negative
10	Abortion	42	1301.0	6	II	5-FU+KSM^*^4	4	2	39	hCG negative
11	Full-term birth	144	354.2	8	I	EMA/CO^*^4	4	2	91	hCG negative
12	Hydatidiform mole	37	4542.0	9	I	EMA/CO^*^7, recurrence occurred 14 months later, EMA/CO^*^3, laparoscopic total hysterectomy was performed after the hCG had decreased to normal, followed by EMA/CO^*^3	7	3	23	hCG negative
13	Hydatidiform mole	123	17285.2	10	III	EMACO^*^4	4	2	57	hCG negative
14	Full-term birth	34	4328.0	8	III	5FU+KSM^*^3, EMA/CO^*^5, laparoscopic total hysterectomy with double salpingectomy, followed by EMA/CO^*^4	12	4	34	hCG negative

a hCG, human chorionic gonadotropin;

b FIGO, International Federation of Gynecology and Obstetrics;

c MTX, methotrexate;

d KSM, actinomycin D;

e 5-Fu, 5-fluorouracil;

f EMA/CO, etoposide + methotrexate + actinomycin D/cyclophosphamide + vincristine;

g EMA-EP, etoposide + methotrexate + actinomycin D/etoposide + cisplatin. CT scan was performed to confirm metastatic lesion.

* denotes the courses of the chemotherapy.

### Treatment and prognosis following diagnosis with GTN

Of the 14 patients, 10 received combination chemotherapy and 4 received monotherapy. In the combined treatment group, the chemotherapy regimen was 5-fluorouracil and actinomycin D (5FU+KSM) in 4 patients, among which 1 patient changed regimen due to drug resistance, and EMA/CO in 6 patients. The median number of chemotherapy courses was 7.5 (4–12 courses), and that of consolidation courses was 2 (1–4 courses). Four patients received a total hysterectomy, and one underwent uterine lesion resection. Nine patients achieved complete remission (CR). One patient with liver and spleen metastasis gave up treatment after her serum hCG level dropped to 75.7 mIU/mL, which returned to normal 2 weeks later. Four patients received MTX monotherapy, and 1 patient achieved CR after initial treatment. Three patients changed regimen due to drug resistance, and all achieved CR. The median follow-up time was 84 months (23–102 months), and all 14 patients survived.

## Discussion

The most common GTD lesions occur in the uterus, and primary lesions outside the uterus are rare and occur in the fallopian tubes, accounting for 0.2–0.8% of cases (5, 6). GTN secondary to hydatidiform mole is relatively easy to diagnose by monitoring serum hCG levels, especially if the interval is shorter. GTN secondary to non-hydatidiform pregnancy, especially without uterine lesions, is more likely to be misdiagnosed. HCG level plateau for 4 consecutive values over 3 weeks or rise ≥10% for 3 values over 2 weeks after hydatidiform mole evacuation indicate GTN diagnosis (7). HCG level normalized within 14 weeks after evacuation in 95% of these patients (8). GTN occurring after hCG normalization is a rare event, which increases the difficulty of diagnosis in these patients.

Studies have shown that the sensitivity and specificity of serum hCG levels combined with transvaginal ultrasound imaging in diagnosing ectopic pregnancy is 97 and 95%, respectively (9). At a serum hCG level of 1,000–2,000 mIU /mL, evidence of ectopic pregnancy can be found in most vaginal ultrasound images (4, 10). At a serum hCG level >3,000 mIU/mL, most intrauterine pregnancy sacs can be detected by vaginal ultrasonography (11, 12). Some scholars have recommended a cut-off value for serum hCG at 3,500 mIU/mL to avoid misdiagnosis or termination of a pregnancy that may be normal (4, 9). If the serum hCG is greater than this level and no pregnancy sac is found by vaginal ultrasound, intrauterine pregnancy is extremely rare. Patients with a positive hCG but no imaging evidence confirming intra- or extrauterine pregnancy should be followed up closely if their hemodynamics are stable (13). If the serum hCG is >3,500 mIU/mL and no evidence of intrauterine pregnancy is found, GTN or other hCG-secreting tumors should be considered. In the present study, there were 7 patients with vaginal color doppler ultrasonography indicating no obvious intra- or extrauterine abnormalities, four of whom had a serum hCG level >2,000 mIU/mL, essentially excluding ectopic pregnancy. Two patients had a serum hCG level >10,000 mIU/mL; therefore, GTN or other hCG-secreting tumors should be considered. The other 2 cases had a serum hCG level <1,000 mIU/mL and should be followed up closely.

Difficult diagnosis by ultrasound imaging in some patients can be assisted by magnetic resonance imaging (MRI) (14). In addition, since most GTN patients have lung metastasis, chest radiographs and lung CT examination can also be used as a differentiator in suspected GTN patients. In some patients with differential diagnosis difficulties, laparoscopy or hysteroscopy is necessary to confirm the diagnosis (15).

Ultrasound image is important criteria for diagnosis of EP, hCG positive and uterine cavity empty, the diagnosis of ectopic pregnancy is usually indicated. However, as hCG is at a low level, it is not casually to make a diagnosis when the condition is stable. HCG and ultrasound images should be closely monitored to avoid misdiagnosis and missed diagnosis. While the hCG level is at a high level, a comprehensive assessment should be make, especially searching for metastatic lesion. HCG positive and metastatic lesion indicate diagnosis of GTN.

The low incidence and non-specific clinical manifestations of GTN are the main reasons for misdiagnosis. GTN is sensitive to chemotherapy, and the vast majority of patients can be cured. It has been reported that the prognosis of GTN with extrauterine lesions is still good (5); however, hematogenous metastasis is the main transfer route of this disease. If distant metastasis such as liver or brain occurs, the prognosis is poor; thus, early diagnosis and treatment should be carried out to avoid delay.

## Data availability statement

The original contributions presented in the study are included in the article/supplementary material, further inquiries can be directed to the corresponding author/s.

## Ethics statement

The present study was approved by the Ethics Committee of the West China Second Hospital of Sichuan University. Informed consent was obtained from all patients.

## Author contributions

PX: data collecting and manuscript writing. TG: data collecting. RY: supervision manuscript review and editing. All authors have read and approved the final manuscript.

## Funding

This work was supported by The Key Project of Sichuan Provincial Department of Science and Technology, Study on the key factors affecting the diagnosis and treatment of major diseases in obstetrics and gynecology (19ZDYF) (Approved by Medical Ethics Committee of West China Second University Hospital, Sichuan University. Ethical Lot Number: 20200076).

## Conflict of interest

The authors declare that the research was conducted in the absence of any commercial or financial relationships that could be construed as a potential conflict of interest.

## Publisher's note

All claims expressed in this article are solely those of the authors and do not necessarily represent those of their affiliated organizations, or those of the publisher, the editors and the reviewers. Any product that may be evaluated in this article, or claim that may be made by its manufacturer, is not guaranteed or endorsed by the publisher.
